# Green and facile synthesis of water-soluble carbon dots from ethanolic shallot extract for chromium ion sensing in milk, fruit juices, and wastewater samples†

**DOI:** 10.1039/d0ra03101a

**Published:** 2020-05-29

**Authors:** Chinawooth Sakaew, Phitchan Sricharoen, Nunticha Limchoowong, Prawit Nuengmatcha, Chunyapuk Kukusamude, Supalak Kongsri, Saksit Chanthai

**Affiliations:** Materials Chemistry Research Center, Department of Chemistry and Center of Excellence for Innovation in Chemistry, Faculty of Science, Khon Kaen University Khon Kaen 40002 Thailand sakcha2@kku.ac.th; Preclinical Science Center, Faculty of Dentistry, Bangkokthonburi University 16/10 Thawi Watthana Bangkok 10170 Thailand; Nuclear Research and Development Division, Thailand Institute of Nuclear Technology (Public Organization) 9/9 Moo 7, Tambon Saimoon Ongkharak Nakhon Nayok 26120 Thailand; Department of Chemistry, Faculty of Science, Srinakharinwirot University Bangkok 10110 Thailand nuntichoo@gmail.com; Nanomaterials Chemistry Research Unit, Department of Chemistry, Faculty of Science and Technology, Nakhon Si Thammarat Rajabhat University Nakhon Si Thammarat 80280 Thailand

## Abstract

Self-functionalized carbon dots (CDs) were prepared from ethanolic shallot extract to obtain a total phenolic precursor. The total phenolic extract was then heated at 180 °C for four hours in an autoclave. Only 1 mg L^−1^ of CDs had high fluorescence emission at 430 nm after excitation at 340 nm and manifested a high selectivity for Cr(vi) ions. The inter- and intra-day emission stability, pH, ionic strength, solvent effect, Stern–Volmer constant, incubation time, speciation of Cr(iii) and Cr(vi) ions, and ion selectivity of the as-prepared CDs were investigated in detail. The proposed method was validated in 20–100 μM linearity with *y* = 2.2346*x* as the set-zero intercept linear equation, 0.9981 as the correlation coefficient, 3.5 μM as the limit of detection (LOD), 11.7 μM as the limit of quantification (LOQ), and 2.78% and 5.29% as the intra-day and inter-day relative standard deviations (RSD), respectively. The recovery of drinking water, milk, soymilk, fruit juices (apple and coconut), tap water, and chromium-coated industrial waste water by the investigated Cr sensor was found to be 78.58–119.69%. Therefore, the proposed Cr(vi) sensor had superior advantages of sensitivity, selectivity, rapidity, and reproducibility.

## Abstract 2

Self-functionalized carbon dots (CDs) were prepared from ethanolic shallot extract to obtain a total phenolic precursor.

## Graphical abstract

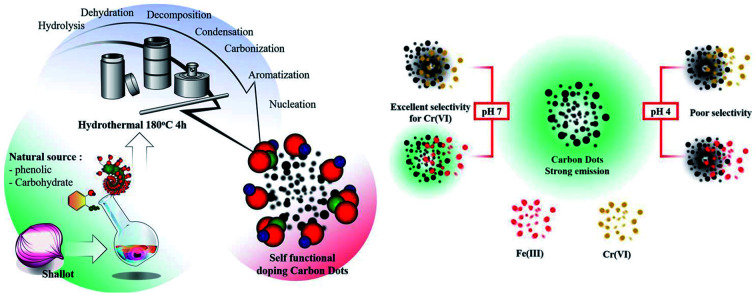

## Introduction

Waste recycling is imperative to hinder the serious threats of human activities to the environment. Household waste, domestic waste, or residential waste can be classified as solid waste comprising garbage and rubbish, such as bottles, cans, clothing, compost, disposables, food packaging, food scraps, newspaper, magazines, and yard trimmings. Solid waste also contains hazardous household waste that enters the water, air, and soil.^1^ The biohazardous waste can cause serious health issues in humans when it is transferred *via* contaminated food. In addition, the contamination can cause air pollution that impacts the respiratory system. Water and soil pollution can be detrimental to agriculture produce, and the toxic compounds are transferred to human *via* food chain. Even so, the wastes still have some concealed benefits usefully.^2^ Solid waste contains carbohydrates, protein, lipid, and phytochemicals; hence, after separation, these chemicals can be reused.

Moreover, terpenoids, alkaloids, sulfur-containing compounds, and phenolic compounds contain heteroatoms in food scraps from a plant.^3^ According to the chemical structures of carbon and plant metabolites, sp^2^ and sp^3^ carbon arrays are tailored to create different nanostructures, such as carbon nanotubes, fullerenes, graphene, and diamond.^4^ Diamond possesses an sp^3^ carbon structure and is generally used in jewellery and cutting works. Fullerenes, nanotubes, and graphene possess an sp^2^ carbon structure and are often applied to conductors, semiconductors, solar cells, heated insulators, touch screens, luminescence cell labelers, biosensor, and chemical sensors.^5^

Carbon also has several derivative structures, such as carbon fibers, onions, rods, and carbon dots (CDs), which are generally used for sensing applications. CDs as new zero-dimensional carbon nanomaterials have drawn much attention due to their positive features, including ease of synthesis, low toxicity, excellent fluorescence properties, good photochemical stability, and high application potential displayed in biomedicine, life science and analytical chemistry. Liu *et al.* developed a unique method for the preparation of CDs from spinach and this nanocomposite was used as a new nanomedicine with dual functions in diagnosis and treatment of tumors.^6^ Garlic and ginger were used to prepare green and low toxic biomass-based quantum dots co-doped with sulfur and nitrogen (S,N-BQDs) for the detection of thrombin.^7^ Hu *et al.* preparation of S and N co-doped CDs from water chestnut and onion for the quantification and imaging of coenzyme A.^8^ The facile and green synthesis of CDs from natural sources has attracted considerable attention synthesized green luminescent water-soluble oxygenous carbon dots with an average size of 3 nm by heating banana juice at 150 °C for four hours without any surface passivating and oxidizing agent.^9^ Hydrolysis was carried out using the existing carbohydrate in the juice as the natural carbon source, and CDs were produced by aromatization and carbonization. Ensafi *et al.* proposed a green strategy to synthesize CDs from saffron successfully.^10^ A quantum yield of 23.6% was obtained by the hydrothermal route. The as-synthesized CDs had high fluorescence intensity, water solubility, and applied to drug sensing and cell imaging. CDs were prepared through dehydration, decomposition, condensation, and aromatization in a Teflon-lined autoclave. Xu *et al.* prepared nitrogen-doped carbon nanodots (N-CDs) through the one-step hydrothermal synthesis route.^11^ Tribute chrysanthemum, a carbon and nitrogen precursor for N-CDs, was produced during the hydrothermal reaction in a Teflon-lined autoclave at 180 °C for 24 h. In addition, N-CDs were used to sense Fe(iii) ions and hydrazine through the turn-off and turn-on mechanisms, respectively. Liu *et al.* found that CDs prepared by the hydrothermal treatment of rose-hearth radish had high fluorescence quantum yield (13.6%), excellent biocompatibility, low-toxicity, and satisfactory chemical stability.^12^ The as-prepared CDs strongly responded to Fe(iii) ions and caused fluorescence quenching. According to the quenching mechanism, lone pair electrons in CDs interact to the incompletely filled d-orbital of the metal ion with the spacing between CDs and reduce metal ions. It induces the energy transfer from CDs to metal ion quencher. Wang *et al.* synthesized N-CDs from *m*-aminobenzoic acid in a Teflon-lined autoclave at 180 °C for 12 h. The as-prepared N-CDs were applied to sense Fe(iii) ions through the quenching mechanism.^13^ Vandarkuzhali *et al.* synthesized CDs from banana pseudo-stem through a hydrothermal route at 120 °C for two hours. The as-synthesized CDs were applied to Fe(iii) turn-off and thiosulfate turn-on sensors.^14^

Hexavalent chromium ions available in the soil, air, and water are toxic and carcinogenic. The environmental remediation and determination of these ions are necessary to prevent the contamination of soil, groundwater, sediment, and surface water. In natural food sources, the probability of Cr(vi) contamination is high. Trivalent chromium is a trace mineral that is essential to human nutrition. However, a large amount of trivalent chromium causes a risk of genotoxicity and prevents normal metabolism and cell functions.^15,16^

In the current work, shallot was used as the natural raw material for CDs synthesis. The concentrated shallot extract was heated at 180 °C for four hours in a Teflon-lined autoclave. The properties of CDs were investigated by FTIR, UV-Vis, PL, SEM, and EDX. The as-developed sensor was used to detect Cr(vi) and Cr(iii) ions.

## Materials and methods

### Chemicals and reagents

All chemicals used in the present study were of the analytical grade. Quinine hemisulfate salt, ethylenediaminetetraacetic acid, mercury(ii) nitrate, lead(ii) nitrate, cobalt(ii) nitrate hexahydrate, zinc(ii) nitrate hexahydrate, copper(ii) nitrate trihydrate, and cadmium(ii) nitrate tetrahydrate were purchased from Sigma-Aldrich, Germany. Sodium phosphate monobasic, disodium phosphate, sodium acetate, sodium chloride, calcium(ii) nitrate tetrahydrate, iron(iii) chloride hexahydrate, and iron(ii) sulfate were obtained from QReC™, New Zealand. Aluminium(iii) chloride, manganese(ii) sulfate monohydrate, nickel(ii) chloride, silver(i) chloride, and magnesium chloride were procured from Carlo Erba, Italy. Chromium(iii) nitrate and potassium dichromate were purchased from Acros Organics™, USA. Acetic acid was supplied by Merck, Germany. Hydrogen peroxide was purchased from Ajax Finechem, Australia. All food samples and shallot were obtained from the local market of Thailand.

### Instruments

An RF-5301PC spectrofluorometer (Shimadzu, Japan) was used as the fluorescence recorder. An Agilent 8453 UV-Visible spectrophotometer (Germany) was used as the absorption recorder. A UB-10 UltraBasic pH meter (Denver, USA) was used as the solution pH buffering controller. A TENSOR 27 attenuated total reflectance-Fourier-transform infrared system (Bruker, Germany) was used for infrared spectroscopy measurements.

### Synthesis of CDs

CDs were synthesized from shallot extract by a hydrothermal route. Shallot was first physically digested by a blender. Freshly-blended shallot (20 g) was sonicated in an ultrasonic bath with 80% ethanol at 0.4 kW and 45 °C. After 30 min, the shallot extract solution was collected. The concentration of the crude extract was calibrated by the total phenolic of standard catechol.

After the successful extraction, the extract (500 mg L^−1^) was placed into a Teflon-line autoclave without any reagent. The hydrothermal synthesis was carried out at 180 °C for four hours (Scheme S1†). A clear yellow solution of CDs was collected after centrifugation for an hour and kept in a refrigerator at 4 °C.

### Fluorescence measurement

The optical properties of CDs (1 mg L^−1^) in a buffer solution of pH = 7 were investigated. The UV-Vis absorption/emission of CDs was examined in a UV-lighting box in the wavelength range of 315–380 nm. The absorption and emission of CDs were measured by an Agilent 8453 UV-Visible spectrophotometer and an RF-5301PC spectrofluorometer (Shimadzu), respectively.

### Quantum yield calculation

The quantum efficiency of CDs was calculated at different concentrations (0.5, 0.75, 1, 1.25 and 1.5 mg L^−1^). The absorption and fluorescence intensity of CDs at 340 nm and 430 nm, respectively, were compared with the absorption and fluorescence intensity of quinine sulfate at 350 nm and 450 nm, respectively.*Q*_c_ = (*Q*_r_)(*I*_c_/*I*_r_)(*A*_r_/*A*_c_)(*η*_c_^2^/*η*_r_^2^)where *Q* is the quantum yield, *I* is the fluorescence intensity, *A* is the absorbance at the excitation wavelength, and *η* is the reflective index of the solvent (quinine sulfate in 0.1 mol L^−1^ H_2_SO_4_ as the reference solution (quantum yield = 54 and *η* = 1.33) and CDs in ultrapure water (*η* = 1.33)). The subscriptions ‘c’ and ‘r’ denote CDs and the reference solution.^17^

### Sensing procedure

The sensing of Cr(iii) and Cr(vi) ions was executed in a 0.05 mol L^−1^ phosphate buffer with pH = 7 at room temperature. The model solution was prepared by placing the calculated amounts of buffer, Cr ions, H_2_O_2_, and CDs into a test tube and mixing them thoroughly before the measurement. After 30 min, the fluorescence emission spectra of Cr ions were recorded in the wavelength range of 400–500 nm under the excitation wavelength of 340 nm. The emission spectra of other metal ions, such as Al(iii), Mn(ii), Hg(ii), Ca(ii), Ni(ii), Pb(ii), Co(ii), Zn(ii), Fe(iii), Fe(ii), Ag(i), Mg(ii), Cu(ii), and Cd(ii) were also conducted to confirm the high selectivity for Cr ions.

All milk samples were pre-treated by 0.05 M HCl and heated at 80 °C for an hour. After the pre-treatment, casein was separated from milk serum. The suspension was filtered to obtain a clear solution, which was stored at 4 °C for further analysis.

## Results and discussion

### Characterization of CDs

**Fig. 1:**
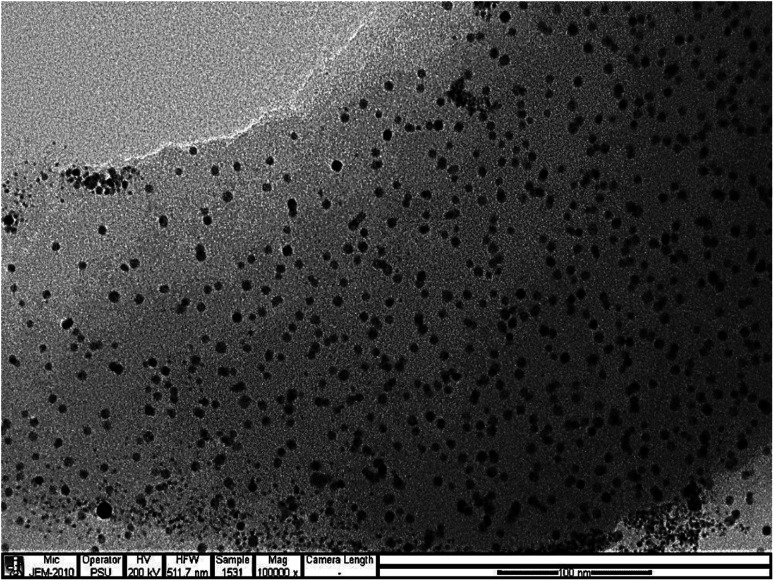
TEM image of CDs.

The morphology and sizes of the CDs was shown in Fig. 1, It can be seen that the product synthesized is composed of very tiny particles and the size distribution based on counting 100 nanoparticles of CDs were mainly distributed in the range of 3.65–8.15 nm with an average size of 4.14 nm. Fig. S1† displays the FTIR spectrum of the as-synthesized CDs. The broad peak at 3300 cm^−1^ corresponds to O–H or N–H vibrational stretching. The weak band at 2900 cm^−1^ appeared due to the stretching vibration of C–H. The medium peaks at 1700 cm^−1^ and 1650 cm^−1^ signifies the stretching vibration of C=O and C–O or N–H, respectively, whereas the peak at 1400 cm^−1^ corresponds to C–N stretching vibration. The medium peak at 1560 cm^−1^ corresponds to C=C vibration. The peak at 1060 cm^−1^ originated from the bending vibration of C–O in carboxyl groups.^18^ The IR band in the wavelength range of 1000–1400 cm^−1^ can be attributed to the stretching vibration of C–O, C–S, and C–H,^19^ this result confirms the existence of multiple functional groups, such as –OH, –COOH, and –NH, in CDs. The EDX spectrum in Fig. S2† and the scanning electron microscopy (SEM) image in Fig. S3† reveals the existence of C (0.277 keV), N (0.392 keV), O (0.525 keV), and S (2.31 keV) in CDs. Si appeared during sample drying. Au appeared from the gold coating. Cl, K, and Ca appeared from the shallot extract.^20^

### Optical properties of CDs

**Fig. 2:**
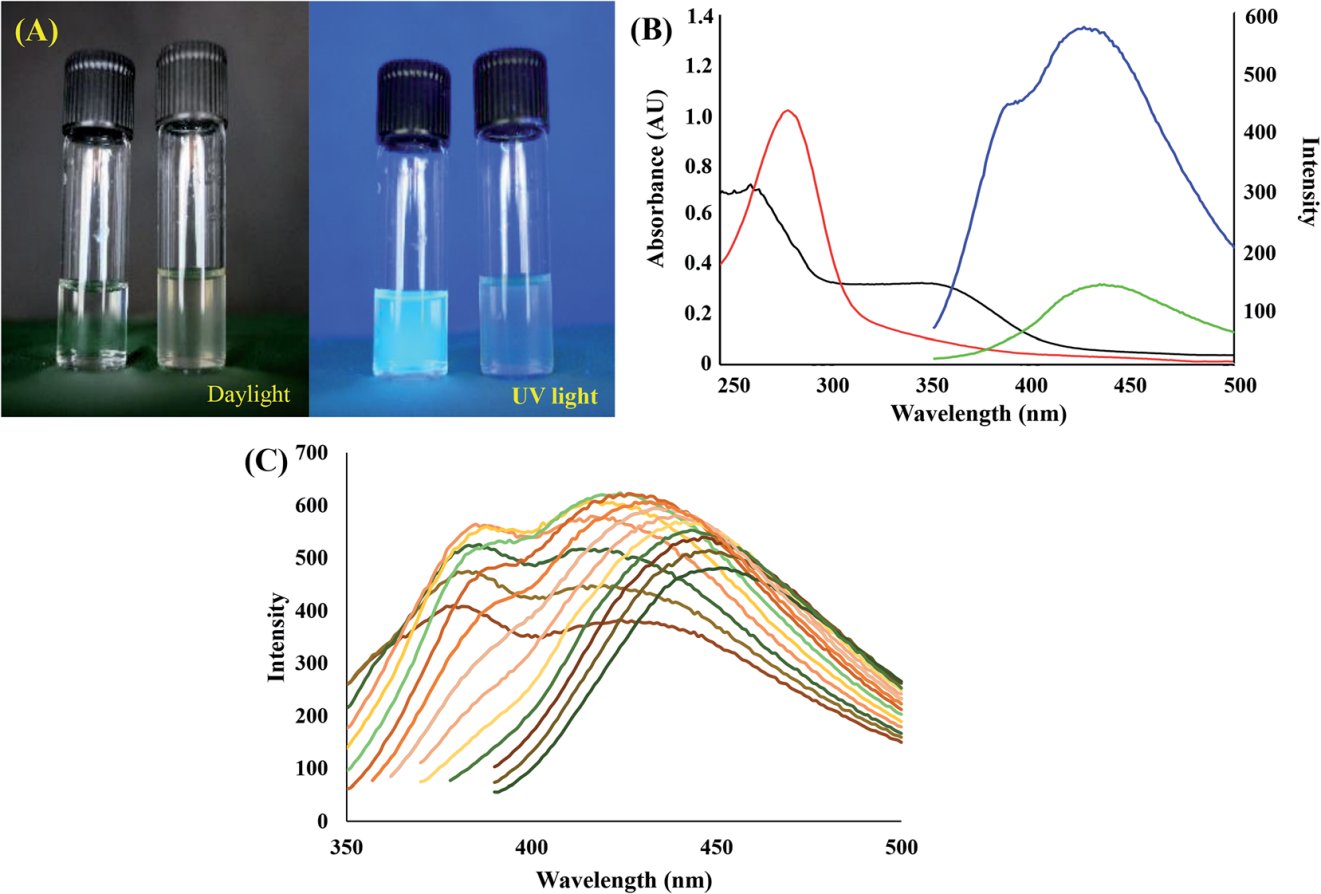
(A) Images of CDs and shallot extract under day light (left) and UV light (right). (B) UV-Visible absorption spectra of CDs (red) & shallot (black) and fluorescence emission spectra of CDs (blue) and shallot extract (green). (C) Fluorescence emission spectra of CDs after various excitation wavelengths.

Fig. 2(A) displays the photograph of as-synthesized CDs under daylight (left) and UV light (right). The brighter emission of the CDs solution demonstrates the successful synthesis of CDs.^21^ The absorption and emission of CDs at 340 nm and 430 nm, respectively confirm the light emission. In Fig. 2(B), the absorbance regions at 250–500 nm and 280–350 nm presents π–π* and n–π* electronic transitions respectively (they both occurred in conjugated C=C and C=O bonds of CDs).^22^ When the excitation varied from 310 nm to 380 nm, the fluorescence experienced a redshift of the maximum emission wavelength along (Fig. 2(C)). The excitation-tunable emission due to the size distribution effect is the inherent property of CDs. The sensitivity of CDs remained constant in the range of 0.5–1.5 mg L^−1^. In the present work, the appropriate concentration of CDs was only one mg L^−1^. Fig. S4† confirms a high emission intensity (the quantum efficiency of the fluorescence emission of Cds was expressed in terms of the relative quantum yield (QY) of CDs). The QY of CDs was calculated as 32.34%, whereas the value for quinine sulfate was 54%.^23^

### Optimization of synthesis conditions

The optimal synthesis conditions for CDs in terms of the differential hydrothermal time and synthesis temperature are presented in Fig. S5(A) and (B),† respectively. Fig. S5(A)† expresses that the fluorescence intensity of CDs gradually increased with the increase of the hydrothermal time. The intensity remained constant at 3–5 h and then started to decrease at the 6th hour. It implies that the occurrence of carbonization and aromatization produced CDs of different sizes.^24^ Hence, the optimal synthesis time was selected as four hours. Fig. S5(B)† reveals that the fluorescence intensity of CDs continuously increased until the highest intensity appeared. Hence, 180 °C was selected as the optimal temperature as the intensity started to decrease between 200 °C and 220 °C.^25^

### Stability of CDs

**Fig. 3:**
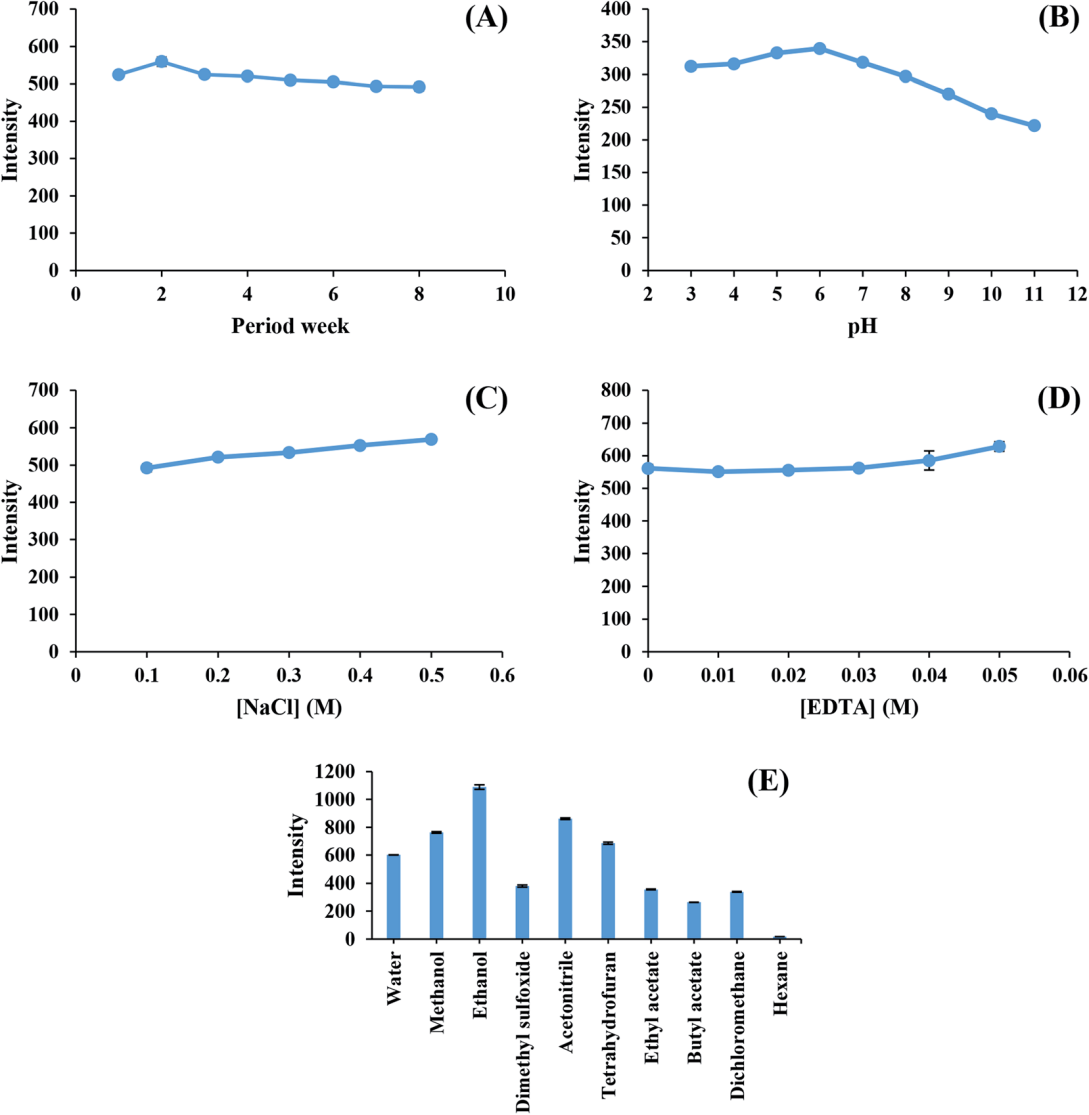
(A) Effect of standing time for 8 weeks on fluorescence intensity of inter-day stability study of CDs. (B) Effect of solution pH on fluorescence intensity of CDs stability. (C) Effect of NaCl concentration on fluorescence intensity of CDs stability. (D) Effect of EDTA concentration on fluorescence intensity of CDs stability study. (E) Effect of different solvents on fluorescence intensity of CDs stability.

The stability of the as-synthesized CDs was studied for eight weeks under 4 °C storage. It is noticeable from Fig. 3(A) that one mg L^−1^ of CDs continuously had a high light emission intensity for eight weeks. The light emission stability of CDs in different pH solutions is presented in Fig. 3(B). The high fluorescence intensity of CDs was detected for the pH range of 3–8. However, the fluorescence intensity of CDs slightly decreased in the pH range of 9–11, thus causing a change in the functional groups of Cds in acidic and basic solutions.^26^ The emission was not affected by NaCl (0.1–0.5 M), and EDTA (0–0.05 M) salts during the study of the ionic strength (Fig. 3(C)) and masking solution effect (Fig. 3(D)).^27^ Therefore, EDTA assisted masking metal interferent ions without any unwanted effect on the high ionic strength.^28^ The effects of soluble solvents (water, methanol, ethanol, dimethyl sulfoxide, and acetonitrile) and non-soluble solvents (tetrahydrofuran, ethyl acetate, butyl acetate, dichloromethane, and hexane) on the fluorescence intensity of CDs are presented in Fig. 3(E). Solvents that allowed CDs to dissolve clearly demonstrated a high fluorescence intensity, whereas all non-polar solvents had a low fluorescence intensity. The effects of different solvents could be explained by the diffusional ability of CDs in the solutions.^29^ Water was selected due to its environment-friendly characteristics.

### Fluorescence sensor for chromium ions

The Stern–Volmer plot of CDs against the increasing concentration of Cr(vi) ions is displayed in Fig. S6,† where *I*_0_ and *I* are the initial photoluminescence intensities of CDs at 430 nm in the absence and presence of Cr(vi) ions, respectively. The correlation coefficient (*R*^2^) and the *Y*-axis interception were set as 0.9966 and 1, respectively, to calculate *K*_sv_ (Stern–Volmer quenching constant).*F*_0_/*F* = 1 + *K*_sv_[Q]where [Q] is the concentration of the quencher (Cr(vi) ions in the present study).^30^ In the Concentration range of 20–100 μM (trace analysis), the *F*_0_/*F* ratio increased continuously without any change in the slope. In the above trace Cr(vi) concentration range, the dynamic quenching process in the sensor followed the results of the incubation time. Thus the quenching intensity from the sudden measurement was indistinguishable from that of the 90 min measurement. It implies that no absorbed shielding effect occurred due to the formation of complexes.

The sensing strategy of the Cr(vi) turn-off mechanism is presented in Scheme S2.† CDs absorbed the 340 nm light by conjugated double bonds to excite π-electrons in the highest occupied molecular orbital (HOMO) into π*-electrons in the lowest occupied molecular orbital (LUMO) of the chromophore. Electrons then relaxed at the ground state and released some photons due to fluorescence emission at 430 nm (called radiative relaxation).^31^ Self-doping thiol and amino functional groups acted as essential auxochromes to induce some quenchers or enhancers to the chromophore of CDs. At pH = 7, Cr(vi) ions existed close to CDs (close enough for electronic transition; however, not enough for complex formation) absorbed the energy of π*-electrons in the LUMO of CDs to transfer them into the incompletely-filled d-orbital of Cr(vi) ions. Due to the stability of Cr(vi) (3s^2^, 3p^6^, 3d^0^) ions, the formations of coordinated covalent bonds did not occur. At pH = 4, the acidic formation of CDs decreased the potential difference between CDs and Fe(iii) ions, and this phenomenon decreased the LUMO energy close to that of the incompletely filled d-orbital (3d^5^) of Fe(iii) ions. The occurrence of static quenching through complex formation between Fe(iii) ions and lone pair electrons of O in phenolic hydroxyl groups on the edge of S-GQDs had been reported in recent literature.^32^ Both phenomena lead to non-radiative electron/hole recombination annihilation and fluorescence quenching.

### Speciation of chromium ions

**Fig. 4:**
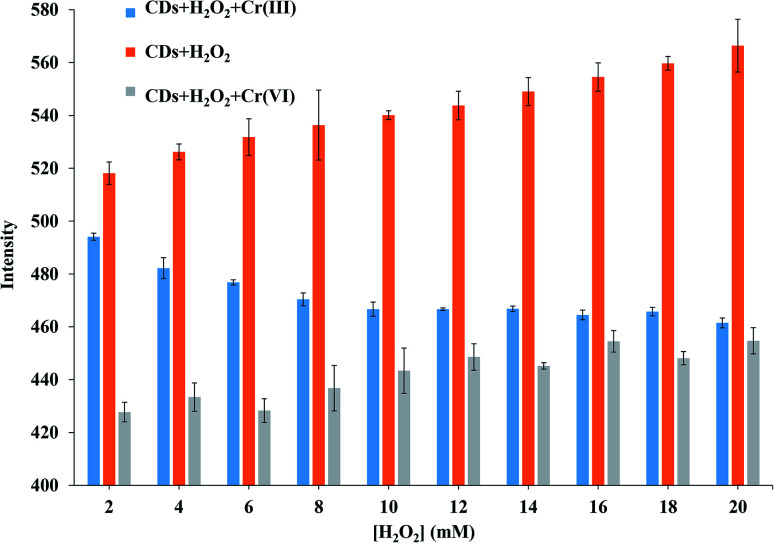
Effect of H_2_O_2_ concentration on fluorescence intensity of Cr(iii) and Cr(vi).

The fluorescence quenching of Cr(vi) ions in a solution of CDs was investigated in detail, and the Cr speciation is displayed in Fig. 4. H_2_O_2_ was used as the strong oxidizing agent to oxidize Cr(iii) ions into Cr(vi) ions at 80 °C for 10 min.2Cr^3+^ + 3H_2_O_2_ + 10OH^−^ → 2CrO_4_^2−^ + 8H_2_O

The above reaction describes the effects of O_2_ from H_2_O_2_ on the fluorescence intensity of CDs and the quenching of produced Cr(vi) ions from Cr(iii) ions.^33^ The fluorescence intensity of CDs slightly increased by H_2_O_2_, thus resulting in enhanced functional stability of CDs. Under a high oxidation state, CDs absorbed more excitation energy and thus had more emission intensity. During the quenching of Cr(vi) ions, the fluorescence intensity of CDs slightly increased. The intensities were slightly decreased at an 80% tall column from the first H_2_O_2_ concentration to 50% from the final concentration. The 20 mM H_2_O_2_ was chosen due to the approximate intensity of the decreased CDs fluorescence from direct Cr(vi) ion quenching and indirect quenching.

### Selectivity

**Fig. 5:**
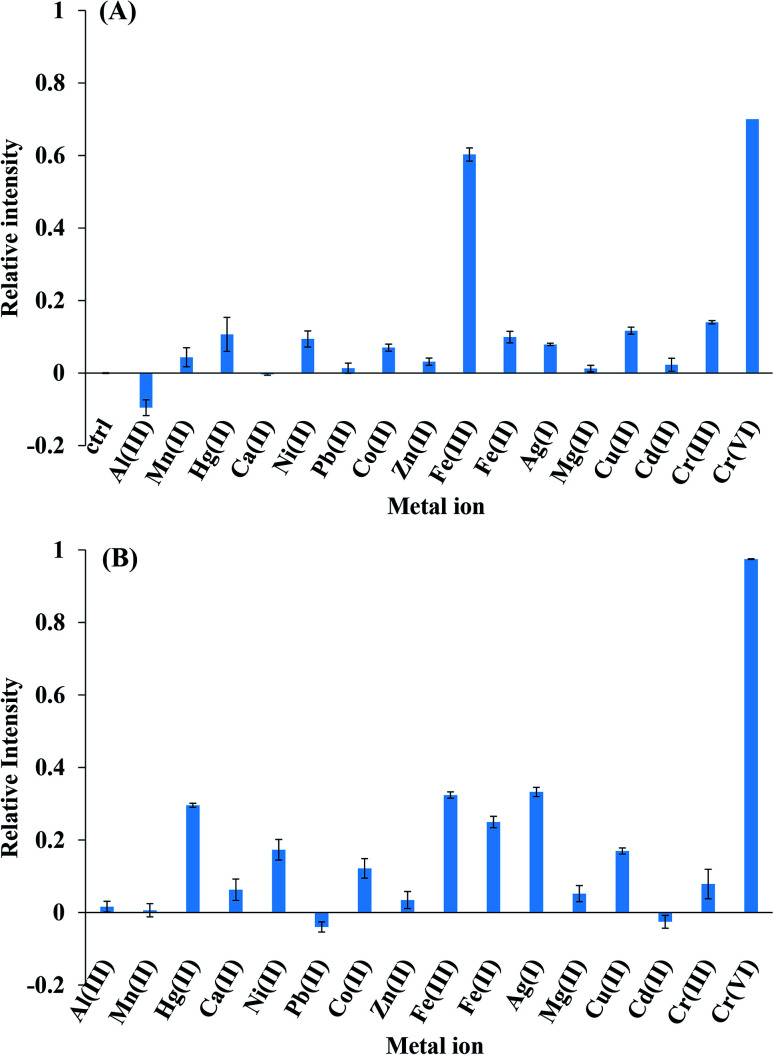
Selectivity study of metal ions on quenching effect of CDs at pH 4 (A) and at pH 7 (B).

Fig. 5 presents the fluorescence intensities of CDs for Al(iii), Mn(ii), Hg(ii), Ca(ii), Ni(ii), Pb(ii), Co(ii), Zn(ii), Fe(iii), Fe(ii), Ag(i), Mg(ii), Cu(ii), Cd(ii), Cr(iii), and Cr(vi). The relative intensity was calculated by the following equation: (*F*_0_ − *F*)/*F*_0_, where *F*_0_ and *F* were initial and after 1 mM metal ion presented fluorescence intensity. It is clear from Fig. 5(A) that Fe(iii) could quench CDs fluorescence for 60% that was as same as the 70% of Cr(vi) quenching. Therefore, it implies that the pH value significantly affected the charged structure of CDs. The charged structure potential increased at pH = 4 and was discounted at pH = 7, thus Cr(vi) ions were selected by the sensor due to the low-charged potential structure of CDs at pH = 7 (Fig. 5(B)). The addition of Cr(vi) ions into CDs resulted in fluorescence quenching, whereas other cations did not affect the intensity of CDs under experimental conditions.

### Method validation

The analytical method was validated under the optimum conditions of several parameters, such as linearity, limit of detection (LOD), limit of quantification (LOQ), and relative standard deviation (RSD). RSD was calculated from the calibration slope of the inter- and intra-day analysis. Table S1† presents the validated terms of the proposed method. The calibration graph followed the linear equation of *y* = *mx* + *b*, where *y* is the relative intensity, *x* is the Cr(vi) concentration, *m* is the slope, and *b* is the *y* interception. LOD and LOQ were defined as 3SD/*m* and 10SD/*m*, respectively, where SD is the standard deviation of Cr(vi) ions with extremely low concentration and *m* is the calibration slope. The evaluation of RSD was carried out by the following expression:% RSD = (SD/*X*-bar) × 100, where SD and *X*-bar are the standard deviation and the average value, respectively. SD and *X*-bar were calculated from the 3 × 3 and 5 × 3 standard preparation slopes for intra-day and inter-day, respectively,^34^ it indicates that the parameters in Table S1† were validated for the repeatability of the analytic strategy.

The working parameters of the investigated probe were compared with previous reported values (Table 1).^35–41^ It is evident that the proposed probe has superior sensitivity and selectivity to existing sensors.

**Table 1:** Carbon quantum dots based sensory probes for chromium detection

Precursor	Synthetic method	Synthesis parameter	Quantum yield	Chromium species	Ref.
Lemon peel waste	Hydrothermal	200 °C, 10 h	14	Cr(vi)	35
Carica papaya waste peels extract	Pyrolysis	200 °C, 15 min	23.7	Total chromium	36
Ethanolamine and 1-carboxyethyl-3-methylimidazolium chloride	Pyrolysis	220 °C, 2 h	21.85	Cr(vi)	37
Tulsi leaves	Hydrothermal	200 °C, 4 h	3.06	Cr(vi)	38
Wool and pig hair	Hydrothermal	240 °C, 6 h	25.6	Cr(vi)	39
Pineapple juice	Hydrothermal	150 °C, 3 h	10.06	Cr(vi)	40
Sucrose and phosphoric acid	Acid carbonization	85 °C, 30 min	0.18	Cr(iii)	41
Shallot	Hydrothermal	180 °C, 4 h	32.34	Total chromium	This work

### Analysis of real samples

The speciation of Cr(iii) and Cr(vi) ions in food and wastewater samples was successfully demonstrated by the proposed method. Table 2 presents the amounts of recovered Cr(iii) and Cr(vi) ions in the samples. The recovery was calculated by the following equation.% Recovery = [(*C*_found_ − *C*_real_)/*C*_added_] × 100where *C*_found_, *C*_real_, and *C*_added_ are the analytical concentration after the addition of the standard, the analytical concentration without the standard, and the known amount of the standard that was spiked in the sample respectively.^42^ The recovery by the proposed method ranged between 78.58% and 119.69% for the mean percentage (*n* = 3) of Cr(iii) and Cr(vi) ions. Therefore, the proposed method yielded a satisfactory result for Cr(iii) and Cr(vi) sensing in real samples.

**Table 2:** Determination of Cr(iii) and Cr(vi) in real samples^a^

Sample	This method					ICP-OES
	Total Cr (μM)	Cr(iii) (μM)	Cr(vi) (μM)	Cr(iii) spiked (% recovery)	Cr(vi) spiked (% recovery)	Total Cr (μM)
Drinking water	n.d.	n.d.	n.d.	107.5 ± 1.66	87.77 ± 2.11	n.d.
Milk 1	n.d.	n.d.	n.d.	89.59 ± 4.75	83.32 ± 4.78	n.d.
Milk 2	n.d.	n.d.	n.d.	78.58 ± 6.87	78.62 ± 7.66	n.d.
Milk 3	n.d.	n.d.	n.d.	82.98 ± 5.83	83.91 ± 6.28	n.d.
Soymilk 1	n.d.	n.d.	n.d.	91.09 ± 3.52	90.69 ± 3.76	n.d.
Soymilk 2	n.d.	n.d.	n.d.	92.64 ± 4.29	99.76 ± 4.39	n.d.
Apple juice	n.d.	n.d.	n.d.	91.71 ± 2.33	95.74 ± 2.39	n.d.
Coconut juice	n.d.	n.d.	n.d.	97.90 ± 2.52	98.01 ± 2.77	n.d.
Industrial wastewater 1	24 ± 1.7	4 ± 2.3	19.9 ± 1.5	107.7 ± 12.91	119.7 ± 13.55	23.4 ± 1.9
Industrial wastewater 2	29.1 ± 5.1	22.4 ± 1.8	6.8 ± 6.1	116.6 ± 14.69	106.5 ± 19.14	29.5 ± 3.4

a n.d.: selected metal ion was not found in the sample used.

The proposed method attained good accuracy during the recovery of Cr(iii) and Cr(vi) ions from clear solutions, such as drinking water, apple juice, and coconut juice. The investigated sensor had a relatively low accuracy during the recovery of Cr(iii) and Cr(vi) ions from all milk samples (1, 2, and 3) due to the interference of lipid and protein. However, the accuracy of the sensor ranged between 100% and 120% during the recovery of Cr(iii) and Cr(vi) ions from all industrial wastewater samples (1 and 2) due to the interference of metal ions. Hence, the recovery by the proposed method ranged between 78.6% and 107.5% for drinking water, milk, soymilk, and fruit juices (apple and coconut). The samples solutions were also analyzed by an inductively couple plasma optical emission spectroscopy (ICP-OES). The results exhibited that the Cr concentration obtained by the proposed sensor compared well with those obtained by the ICP-OES method. The results from this work can be confirmed the reliability of the proposed sensor in real application as well.

## Conclusion

Self-functional CDs were successfully synthesized using shallot as the raw material at 180 °C for four hours under high fluorescence excitation/emission at 340/430 nm. The quantum efficiency of quinine sulfate was found as 32.34%. The stability of CDs was studied by different techniques. The inter-day fluorescence emission stability of CDs continued for eight weeks under 4 °C storage, whereas the intra-day emission stability was noticed for 90 min under the ambient light. The high fluorescence intensity of CDs was detected for the pH range of 3–7. The emission was not affected by NaCl and EDTA salts during the study of the ionic strength and masking solution effect. Water was selected as the solvent of CDs (water-soluble CDs) due to its high emission intensity and eco-friendly characteristics. The sensor for chromium(vi) ions was selected by a dynamic quenching system at one mg L^−1^ CDs for a sudden measurement to 90 min incubation with *K*_sv_ = 1163.4 L mol^−1^ and pH = 7. The speciation of Cr(iii) and Cr(vi) ions was achieved after oxidation at 80 °C for ten min. In addition, the proposed method was validated in 20–100 μM linearity with *y* = 2.2346*x* as the set-zero intercepted linear equation, 0.9981 as the correlation coefficient, 3.5 μM as the limit of detection, 11.7 μM as the limit of quantification, 2.78% and 5.29% as the intra-day and inter-day relative standard deviations respectively. The recovery of drinking water, milk, soymilk, fruit juices (apple and coconut), tap water, and chromium-coated industrial wastewater by the investigated Cr sensor was found as 78.58–119.69%. Therefore, the proposed Cr(vi) sensor had superior advantages of sensitivity, selectivity, rapidity, and reproducibility.

## Conflicts of interest

All authors have none to declare.

## Supplementary Material

RA-010-D0RA03101A-s001
