# Supplementary material for: Green and facile synthesis of water-soluble carbon dots from ethanolic shallot extract for chromium ion sensing in milk, fruit juices, and wastewater samples
Source: RSC Adv. 2020 May 29;10(35):20638–45. doi: 10.1039/d0ra03101a (PMC9054292; doi:10.1039/d0ra03101a)
Supplement: RA-010-D0RA03101A-s001 [file RA-010-D0RA03101A-s001.pdf]

Electronic Supplementary Material (ESI) for RSC Advances.
This journal is © The Royal Society of Chemistry 2020

Supporting information

Green and facile synthesis of water-soluble carbon dots from ethanolic shallot extract for chromium ions sensing in milk, fruit juices, and wastewater samples

Chinawooth Sakaew[1], Phitchan Sricharoen[2,3], Nunticha Limchoowong[4]*, Prawit Nuengmatcha[5], Chunyapuk Kukusamude[3], Supalak Kongsri[3] and Saksit Chanthai[1]**

[1]Materials Chemistry Research Center, Department of Chemistry and Center of Excellence for Innovation in Chemistry, Faculty of Science, Khon Kaen University, Khon Kaen 40002, Thailand

[2]Preclinical Science Center, Faculty of Dentistry, Bangkokthonburi University, 16/10 Thawi Watthana, Bangkok 10170, Thailand

[3]Nuclear Research and Development Division, Thailand Institute of Nuclear Technology (Public Organization), 9/9 Moo 7, Tambon Saimoon, Ongkharak, Nakhon Nayok 26120, Thailand

[4]Department of Chemistry, Faculty of Science, Srinakharinwirot University, Bangkok 10110, Thailand

[5]Nanomaterials Chemistry Research Unit, Department of Chemistry, Faculty of Science and Technology, Nakhon Si Thammarat Rajabhat University, Nakhon Si Thammarat 80280, Thailand

Corresponding author, e-mail address: nuntichoo@gmail.com* (N. Limchoowong), sakcha2@kku.ac.th** (S. Chanthai)

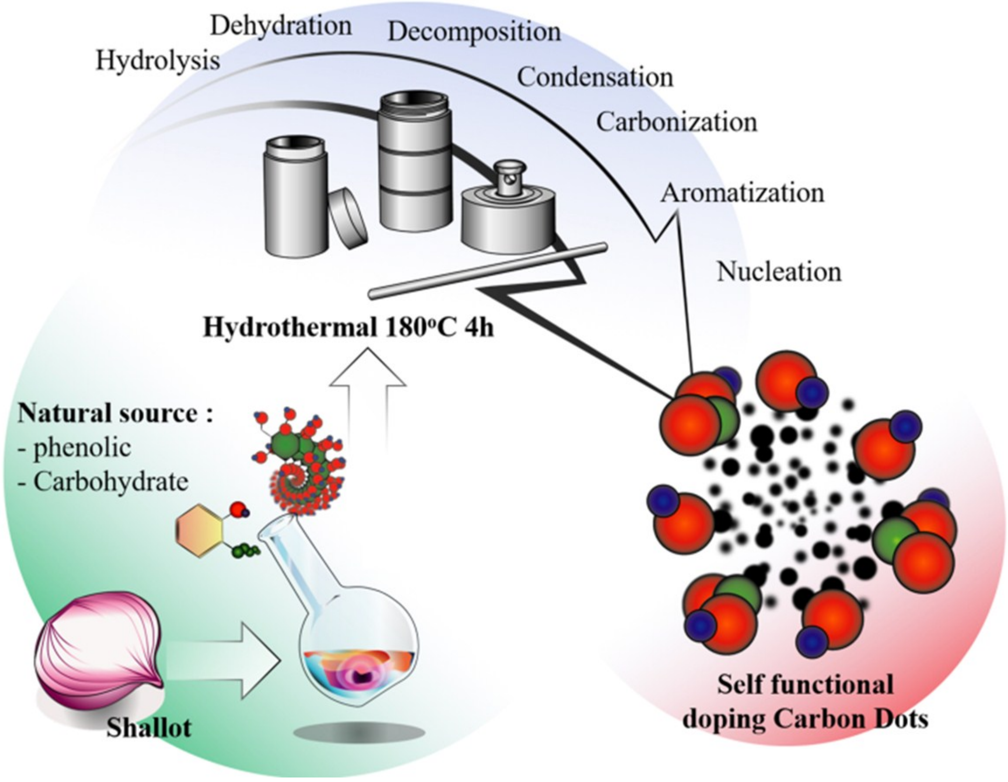

Scheme S1. Extraction and hydrothermal step for CDs synthesis from shallot.

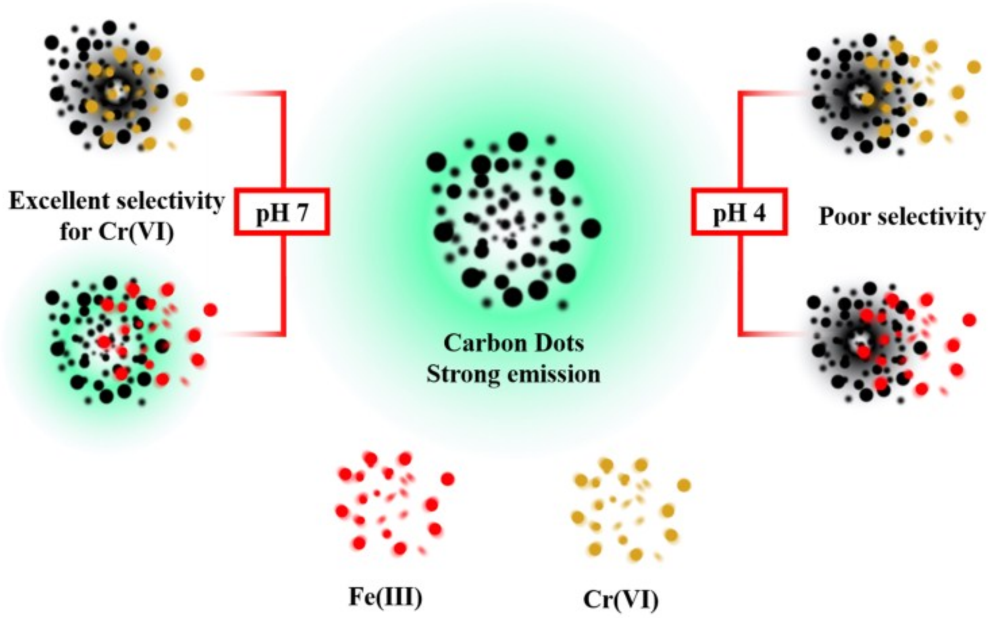

Scheme S2. Quenching mechanisms of Cr(VI) and Fe(III) at pH 4 and pH 7.

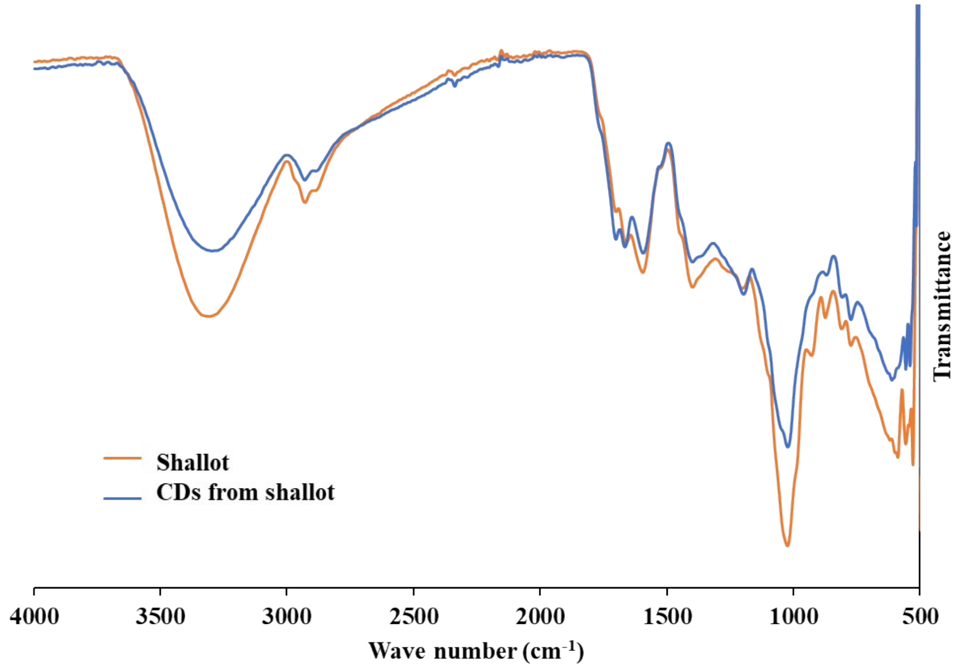

Fig. S1. FTIR spectra of shallot extract and its CDs.

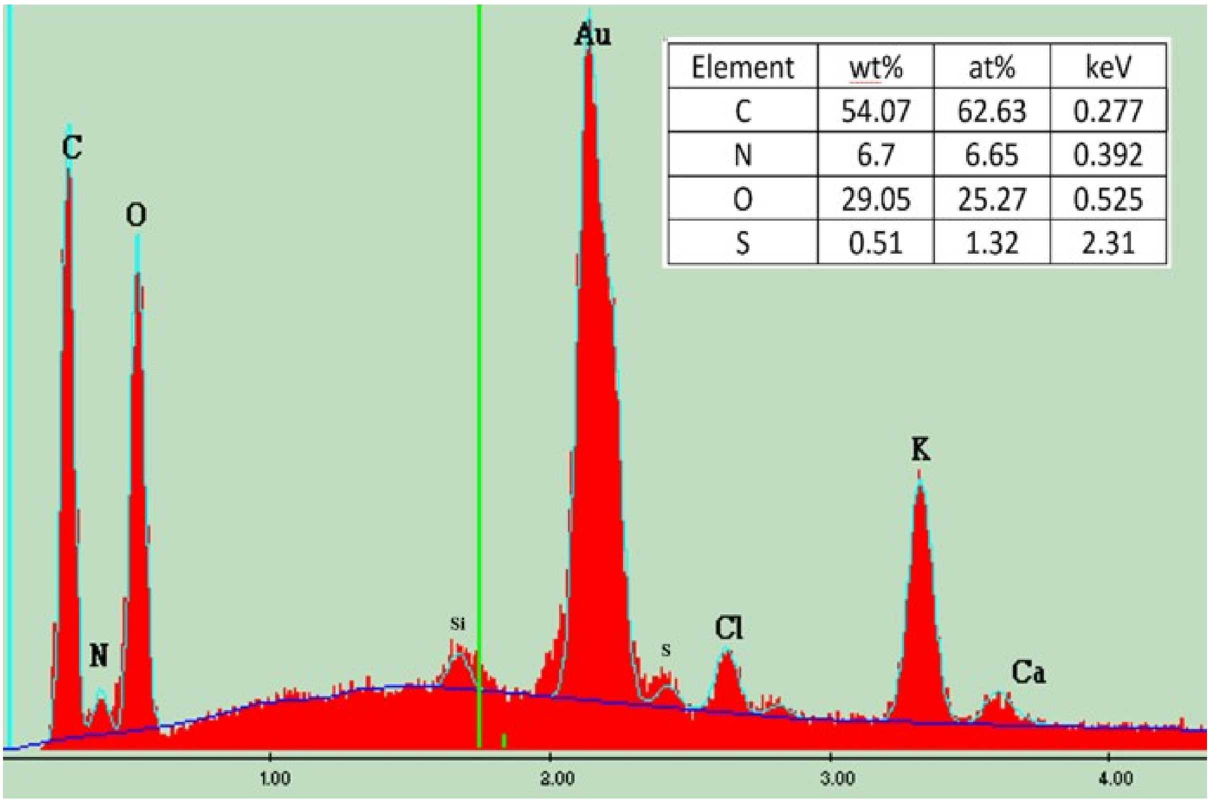

Element	wt%	at%	keV
C	54.07	62.63	0.277
N	6.7	6.65	0.392
O	29.05	25.27	0.525
S	0.51	1.32	2.31

Fig. S2. EDX pattern of CDs.

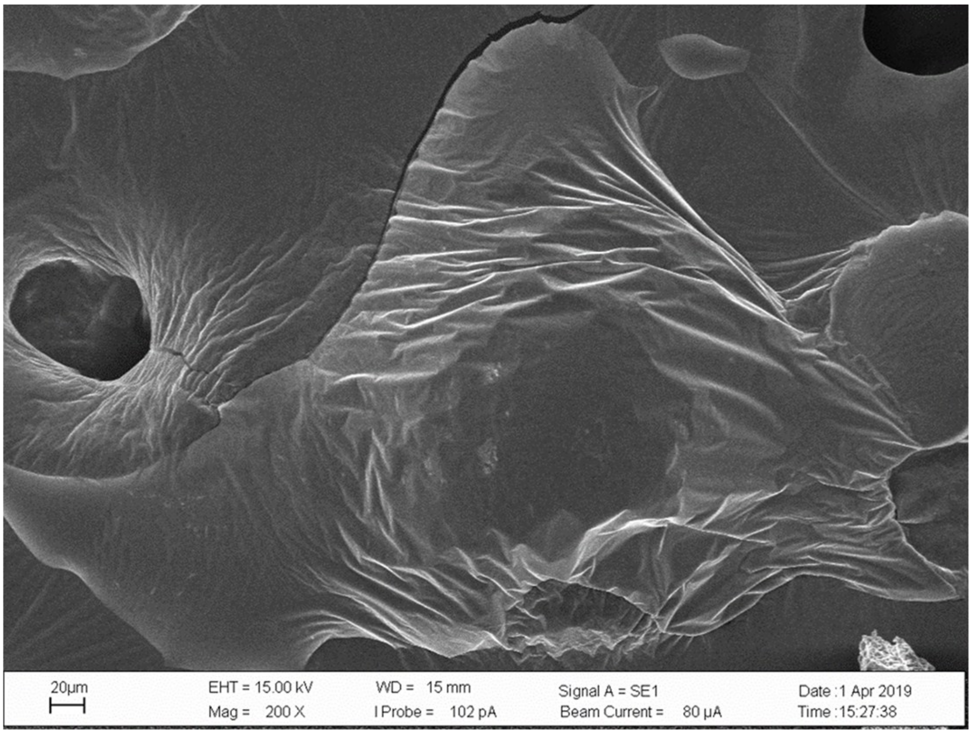

Fig. S3. SEM image of CDs.

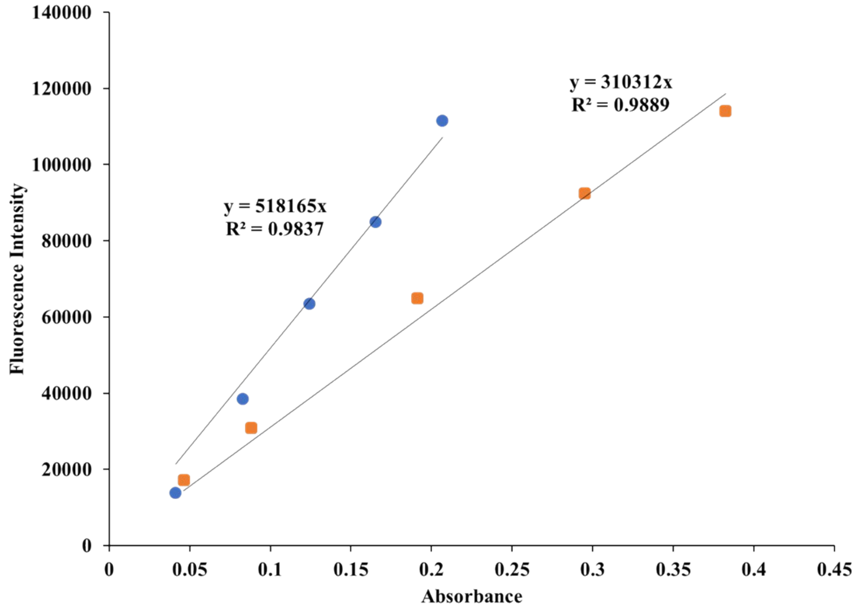

Fig. S4. Relative quantum efficiency of CDs and quinine sulfate.

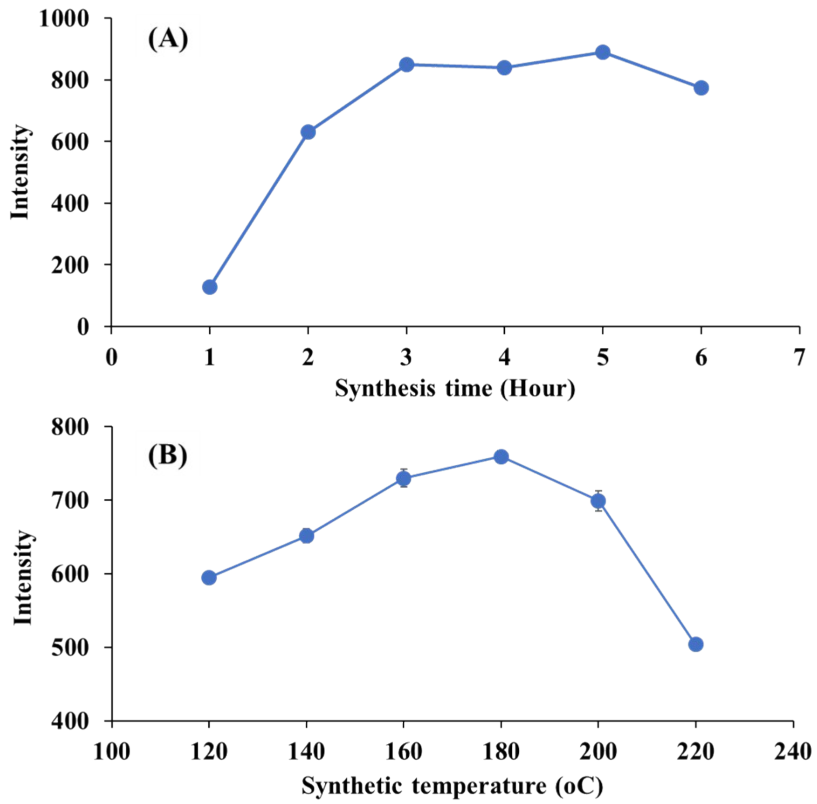

Fig. S5. (A) Effect of incubation time on fluorescence intensity of CDs for hydrothermal step. (B) Effect of incubation temperature on fluorescence intensity of CDs for hydrothermal step.

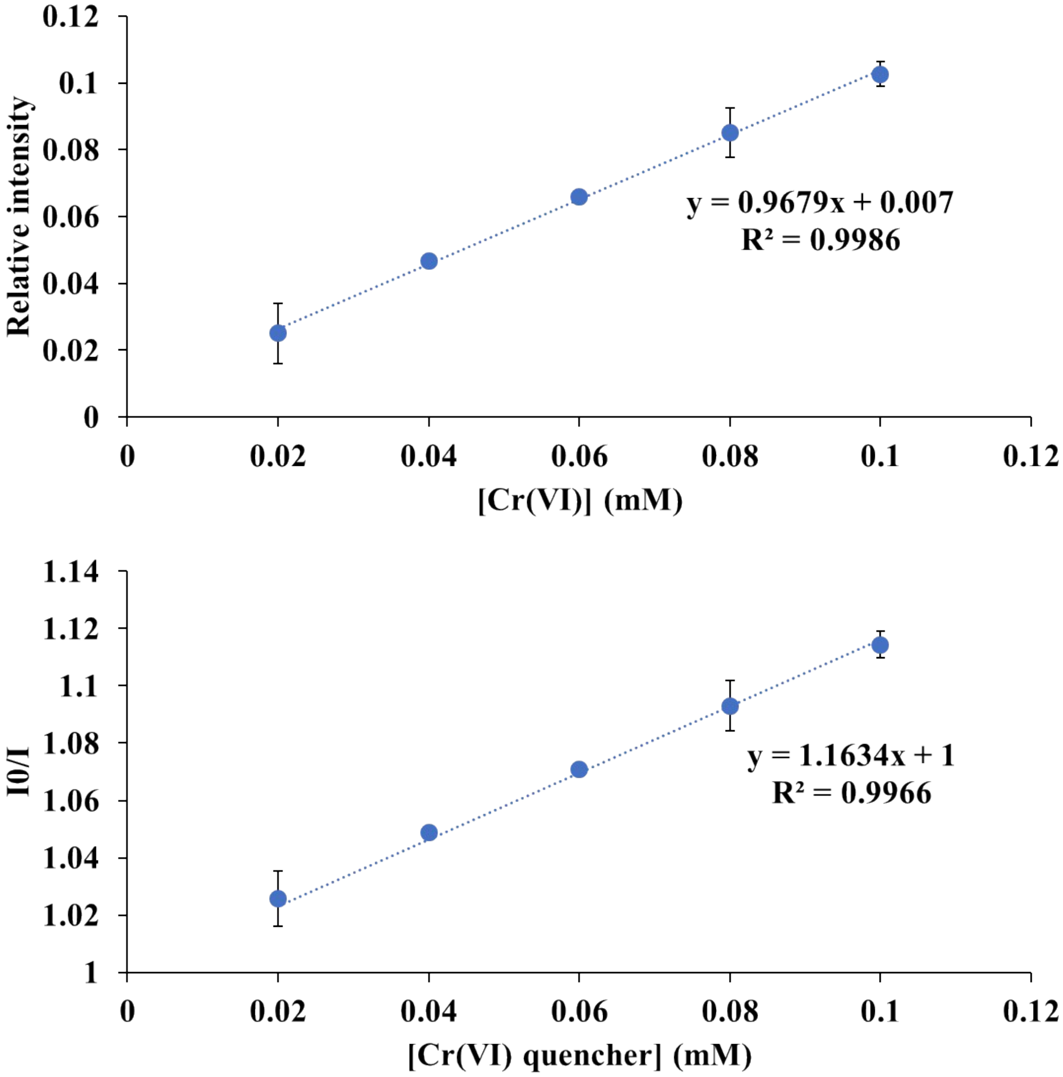

Fig. S6. (A) Effect of various concentrations of Cr(VI) quenching of CDs. (B) The Stern Volmer plot for Cr(VI) quenching effect.

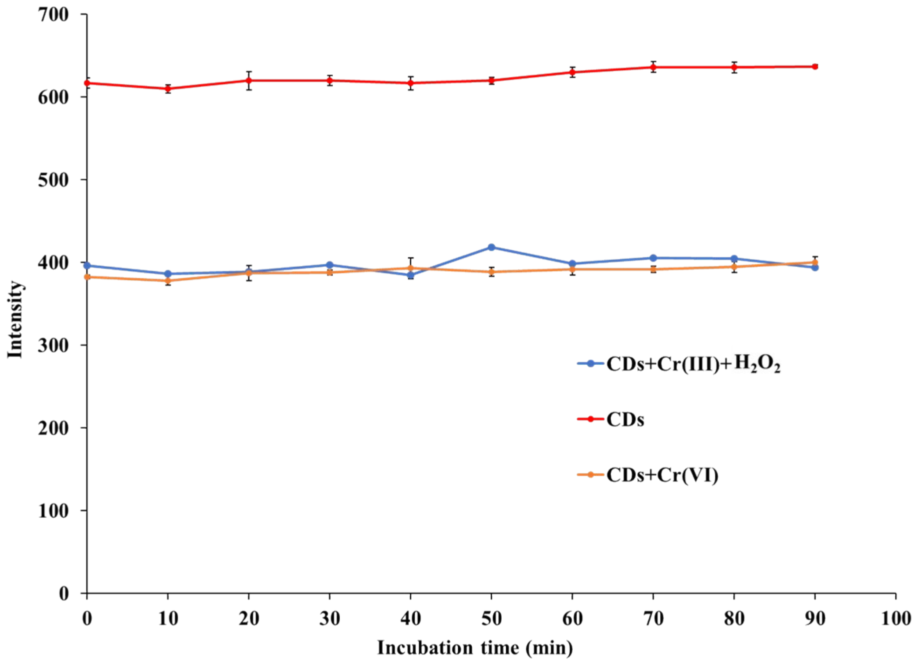

Fig. S7. Effect of incubation time (conversion reaction) of Cr(III) to Cr(VI) by oxidation with H_2O_2

Table S1. Analytical characteristics of the proposed method

Analytical parameter	Analytical feature
Linearity (μM)	20-100
Linear equation ((0,0) intersection) for	
20 - 100 (μM)	y = 2.2346x
100-1000 (μM)	y = 1.0928x
Correlation coefficient (R^2) for	
20-100 (μM)	0.9981
100-1000 (μM)	0.4725
Limit of detection (LOD), (μM), (n = 11)	3.5
Limit of quantification (LOQ), (μM), (n = 11)	11.7
Relative standard deviation (RSD), (%) for	
Intra-day analysis (n = 3x3)	2.78
Inter-day analysis (n = 5x3)	5.29
